# Outcome of a four-hour smoking cessation counselling workshop for medical students

**DOI:** 10.1186/s12971-016-0103-x

**Published:** 2016-11-25

**Authors:** Kurosch Purkabiri, Valentina Steppacher, Kathrin Bernardy, Nikola Karl, Verena Vedder, Michèle Borgmann, Anja Rogausch, Uz Stammberger, Robert Bals, Tobias Raupach, Volker Koellner, Jürg Hamacher

**Affiliations:** 1 Division of Pulmonary Medicine, Internal Medicine, University Hospitals of Homburg/ Saar, Saarland University, Homburg/Saar, Germany; 2 Clinic of Pulmonary Medicine and Respiratory Cell Research, University Hospital Basel, Basel, Switzerland; 3 Division of Pain Medicine, Berufsgenossenschaftliches Universitätsklinikum Bergmannsheil GmbH, Bochum, Germany; 4 Divison of Internal Medicine, Hospital of Münsingen, Münsingen, Switzerland; 5 University Hospital of Psychiatry, Bern, Switzerland; 6 Clinic Sonnenhalde, Riehen, Switzerland; 7 Division of Assessment und Evaluation, Institute for Medical Teaching, University of Bern, Bern, Switzerland; 8 Department of Cardiology and Pneumology, University Hospital Göttingen, Göttingen, Germany; 9 Department of Behavioral Therapy and Psychosomatic Medicine, Rehabilitation Center Seehof, Federal German Pension Agency, Teltow, Germany; 10 Medical Faculty, Saarland University Hospitals, Homburg/Saar, Germany; 11 Clinic of Internal Medicine, Lindenhofspital, Bremgartenstrasse 119, CH-3012 Bern, Switzerland

**Keywords:** Smoking cessation counselling, Medical education, Curriculum, Communication skills, Motivational interviewing, Knowledge, Skills, Attitude, Tobacco cessation education

## Abstract

**Background:**

Lack of smoking cessation education in undergraduate medical training hinders healthcare professionals in providing adequate tobacco cessation counselling.

We developed a comprehensive 4-h smoking cessation counselling course for medical students that is easy to incorporate in a medical school curriculum, and assessed its short-term outcome for knowledge, skills, and attitudes.

**Methods:**

Eighty-eight medical students (53f, 35 m) were educated by a doctoral student in five identical 4-h courses. A 45-min theoretical introduction was followed by patient-physician role-playing by student pairs. Knowledge, skills, and attitude were assessed before and 4 weeks after the course by questionnaires, and by blinded analysis of pre- and post-course videos of a five-minute standardized patient situation.

**Results:**

Knowledge: Before the course 10.6 (mean, SD: 2.7) questions out of 29 were answered correctly, and increased to 19.2 (3.6) after the course (*p <* 0.0005). Major features of the students’ counselling skills improved. Significant and highly relevant attitude changes reflected increased motivation to counselling smokers.

**Conclusion:**

Implementing a four-hour smoking intervention workshop into a medical curriculum was highly effective in improving students’ knowledge, skills and attitudes towards smoking counselling, as well as providing them with additional clinical competencies.

**Electronic supplementary material:**

The online version of this article (doi:10.1186/s12971-016-0103-x) contains supplementary material, which is available to authorized users.

## Background

Smoking is the leading single cause of preventable disease [1] and death in the western world. Smoking-related mortality is estimated to increase from 3 million annually (1995 estimate) to 10 million annually by 2030, with 70% of these deaths occurring in developing countries [2]. The vast majority of these smokers wish to quit, but find it difficult to do so, in large part because of the addictive effects of nicotine. Smoking cessation treatment represents one of the most cost effective healthcare interventions [3].

Effective behavioural and pharmacological treatments, coupled with professional counselling and advice, are required to improve smoking cessation rates [4]. Since smoking duration is the principal risk factor for smoking-related morbidity, the treatment goals should be early cessation and prevention of relapse [4]. Physicians are uniquely placed to assist in smoking cessation as a crucial preventive medical measure: They are generally accessible to the whole community; are frequently visited; are the preferred source of information on health and lifestyle matters; and have been demonstrated to be effective agents of change [5]. Furthermore, they act as visible role models and may unintentionally affect the smoking behaviour of others. Developing attitudes of future physicians towards preventive medicine (e.g. smoking, other substance or non-substance abuse cessation, nutrition counselling/education, weight reduction, physical activity) will likely provide a major impetus for the inclusion of preventive medicine content in medical school curricula and in other forms of physician education [6]. Assuming that physicians’ personal attitude towards the issue of smoking cessation counselling is to a great extent formed during their medical education, any successful tobacco control measures within the medical profession will need to begin prior to graduation from medical school. Teaching modules should focus on the responsibility of physicians in disease prevention and training in specific smoking cessation techniques early in undergraduate curricula [7].

Despite its importance, few medical students receive formal training in smoking cessation counselling [7, 8]. Anxiety and feelings of being ill-prepared for practice are common amongst medical students [9]. Several studies have documented physicians’ failure to advise their patients regarding smoking [10]. Most smoking patients are not instructed or assisted in their cessation efforts. Given the importance of smoking in practically all aspects of medicine and the role of clinicians in advising and aiding cessation, such competence is needed [7].

The purpose of this project was to teach students how to work effectively with patients in the area of smoking cessation counselling through an efficient 4-h comprehensive course. The workshop was designed to be easy to implement within the medical school curriculum. Key competences for adequate and successful smoking cessation counselling were intended to be as effective as possible, but still applicable to other health behaviours within daily clinical practice [11].

## Methods

### Course

A 4-h voluntary extracurricular smoking cessation teaching course “by medical students for medical students” was offered by the medical faculty of Saarland University in Homburg/Saar, Germany. The course consisted of a 45-min PowerPoint presentation as a theoretical introduction, and a short discussion followed by 3 h of physician-patient role-playing and group discussions. Contents were in accordance with the Swiss and international clinical guidelines for smoking cessation, as well as with Swiss smoking cessation teaching courses for physicians [3, 12, 13]. The course included a pre- and a post-course assessment scheduled 4 weeks apart. The optional smoking cessation counselling courses, including full information on assessment, were offered for all medical students. In order to accommodate the large response, the course was run five times between January 2006 and June 2007. The course was conducted by a doctoral student, VS (5th year medical student) and took place in the seminar rooms of the University Hospitals. A medical expert, JH, with 25 years of experience in smoking cessation, including teaching smoking cessation counselling, was the full-time supervisor. Fifteen to 20 1st–6th year medical students attended the course.

A 15-min lunch break was included in the training period. Time was allocated for discussion and questions at the end of the course. After each course all participating students were encouraged to fill out an anonymous course evaluation form in order to optimize future courses. Written informed consent was obtained from all participants in order that the data and film could be used for scientific research as well as for improving the course.

### Study objectives and outcome measurements

The “success” or “partial success” of the course was reflected by “significant” (*p* < 0.05) and “relevant” (d = 0.25) improvements in each of the three dimensions of medical students’ smoking cessation competence (*knowledg*e, *skills*, *attitude* [for definitions: see Additional file 1]) 4 weeks after the course, and were assessed as primary endpoints.

The course was considered “successful” according to our a priori definition whenever all three dimensions of competence (*knowledge, skills, attitude*) had significantly and relevantly improved in the assessment 3–4 weeks after the course ended. The course was considered “partially successful” whenever less than three, but at least one dimension of competence had improved after the course.

### Formats for teaching and educational methods

#### Theoretical in-depth lecture

The doctoral student (VS) delivered a 45-min didactic PowerPoint presentation, containing compact tobacco control information and set priorities to evidence-based practice-oriented theoretical knowledge needed for smoking cessation counselling by physicians. Together with additional literature, this short didactic approach served as a solid, but expandable theoretical basis for smoking cessation intervention training and clinical practice. The slides were based on literature and course materials [4, 11, 13, 14].

#### Physician-patient role-playing

Over a 3-h period, the students participated in role-playing alternating between the smoking patient and the therapist, according to the six “Stages of Change” by Prochaska and DiClemente [15]. Each student was given a description of the case setting corresponding to one of the stages of change. Flip charts containing the most important points for smoking cessation counselling were also distributed, and the course leader explained their optimal use. Participants were encouraged to rate each other’s performance under interpersonal and emotional aspects with the aid of a check list and to discuss each other’s performances after each role-play. Five to ten minutes of interactive group discussions were implemented between each role-play. Both the doctoral student and expert continuously supervised the activities, provided feedback and answered questions.

#### Teaching materials

Participants were given reading assignments from communication, health behaviour change and specialist smoking cessation literature, as well as instructive handouts to guide them through their interviews. The handout-set included 6 items:

1. A counsellor’s reference manual (script) summarizing current knowledge on tobacco use and cessation in clinical practice introducing the six stages of change: “pre-contemplation,” “contemplation,” “preparation,” “action,” “maintenance,” and “relapse,” as outlined by Prochaska and DiClemente [15]. The manual also included a record sheet for consultations with smokers, and to facilitate data management of tobacco use and smoking cessation interventions.
2. A flip chart containing two algorithms summarizing counselling strategies and pharmacological therapy designed for the student’s or physician’s pocket or desk.
3. Separate notes informing the students about each of the different role-playing settings according to the six stages of change by Prochaska and DiClemente [15].
4. A check list for self-assessment after the role-playing containing a list of crucial facts required to counsel smokers.
5. Information about the interview setting: The student were in the role of a general practitioner meeting a patient in order to re-examine a healed fracture of the ankle joint.
6. A set of patient instructions for the use of the discussed nicotine replacement products and smoking cessation drugs (bupropion, varenicline). The handout items are listed in Additional file 2: Table S2.

### Pre- and 4 weeks post-course assessments

#### Questionnaire on medical students’ smoking cessation counselling competence

Students’ smoking cessation intervention competences namely “knowledge,” “skills,” and “attitude” were assessed prior to training (to determine baseline performance levels) and 4 weeks after the course by questionnaires assessing the above-mentioned three dimensions of competence. The questionnaires were developed for this study by an expert panel, using the evidence-based literature of medicine [16] and social sciences combined with empirical experiences in smoking cessation counselling.

##### Assessing “knowledge” and the self-assessment of “attitude” (subjective assessment of “attitude”)

The dimensions of “knowledge” and “self-assessment of attitude” were rated with the aid of a questionnaire containing 41 questions of which 29 multiple choice questions assessed knowledge. The basic knowledge required for efficient smoking cessation counselling is the essential composition of the course content, and can be summarized as: 1) cigarettes and nicotine, 2) diseases associated with smoking, 3) dependency and addiction, including an adapted pharmacological therapy for nicotine dependency, 4) the behaviour-change model with the different stages of change in the progress of smoking cessation according to Prochaska and DiClemente, and 5) related appropriate counselling strategies centred on basic communication concepts.

Twelve questions targeting “attitude” assessed attitudes towards smokers, counselling of smokers, the smoking cessation course and their self-evaluated tolerance towards smokers. All “attitude” items were answered with the aid of a Visual Analog Scale (VAS; scale range: 0-100 units). The comprehensibility as well as the difficulty level of the questions were piloted with medical students not participating in the course.

#### Five minutes of videotaped standardized patient interviews pre- and 4 weeks post-course, allowed an off-line blinded video analysis

Each participant had two (pre- and 4 weeks post-course) brief (5 min) encounters with a standardized patient (trained actor) representing a stage of change (“pre-contemplation”). Before the interview, each student was provided with information about the setting in which the general practitioner meets a patient in order to re-examine a healing fracture of the ankle joint. Both encounters were videotaped for a blinded analysis by two independent observers. The dimension of the contents as well as the patient-physician relationship and interaction were assessed with 33 items using visual analog scales. This allowed “skills” assessment as well as the externally assumed assessment of “attitude” (in contrast to subject’s self-assessed “attitude” in the questionnaires).

##### Assessing “skills” and the externally assumed assessment of “attitude”

Two independent and “blinded” evaluators (a senior medical student and a physician in internship who used this analysis as his doctoral thesis) were recruited and extensively trained individually and as a group, and repeatedly supervised by a multidisciplinary expert panel to rate the videotaped performances [17] using a rating check list. Evaluators were provided with a detailed evaluator’s manual based on criteria of an expert panel of medical practitioners and smoking researchers used in Swiss smoking cessation courses, including a questionnaire assessing whether the student, in the role of a counsellor, covers the most important points of smoking cessation. We assessed whether the student was able to detect the patient’s nicotine dependency, evaluate the smoking history and identify the patient’s stage of change according to Prochaska and DiClemente. Moreover, the check list covered the students’ abilities to focus on the patient’s problems concerning smoking cessation, offer possible treatments including an appropriate pharmacological therapy and suggest further appointments. Details of the rating are indicated in Additional file 3.

Any ambiguity had to be discussed and resolved together with the third evaluator and the expert panel. No information about the time point (pre- or post-course assessment) of the interviews was provided to the evaluators. More information about the structure of the rating schedule can be found in Additional file 4.

### Statistics

Data were analysed with the Systat 12.0 software (Systat Inc., Point Richmond, CA, USA). A p-value of ≤ 0.05 was considered significant. The median and range is given for descriptive statistics of variables with normal distribution of the mean (standard deviation) and for nonparametric distribution of variables. According to group number and design, paired T-test, unpaired T-test, analysis of variance (ANOVA) with planned contrast analysis between groups or Kruskal-Wallis and Mann-Whitney U-test were applied, respectively. For computation of Cohen's *d* effect sizes the Online Calculator for Effect Sizes by Lenhard and Lenhard [18] was used. Cohen's d value (*d*) was used to estimate the effect sizes in the pre-post comparisons of the group. As there are no agreed standards for how to interpret an effect size, the most established approach by Cohen was applied [19, 20]. As a rule of thumb he suggested *d* ≥ 0.2 for small, *d* ≥ 0.5 for moderate and *d* ≥ 0.8 for large-sized effects. Wolf [21] simplified Cohen’s scopes into *d* ≥ 0.25 meaning that the effect is educationally/academically significant (i.e. “something was learned”) and *d* ≥ 0.50 standing for an effect being further practically significant (therapeutic, i.e. “something happened”).

## Results

### Study subjects and biometric data

Eighty-eight (eight pre-clinical and 80 clinical) medical students participated. Twenty percent of the participants were smokers (11 female and five male), 66% non-smokers and 14% former smokers (i.e. more than 100 cigarettes in life).

### Competence dimensions

#### Knowledge

Whereas participants could answer 10.6 (mean, SD: 2.7) questions out of 29 correctly prior to the course, 4 weeks after the course they demonstrated increased knowledge, and answered 19.2 (mean, SD 3.6) questions successfully (*p <* 0.0005) corresponding to 81%. The effect size by Cohen’s d was 2.7, which is considered as an “extremely relevant” improvement. The results are shown in Additional file 5: Figure S1. Objective results were distinctly reflected in students’ subjective anonymously written feedback forms, as well as in verbal feedback.

#### Skills

Significant gains were observed in the investigation of the motivation to quit smoking, in recommendations for stopping smoking, and suggestions for practical strategies to facilitate smoking cessation and prevent relapse. Substantial gains were also noted in the assessment of the duration and amount of tobacco consumption, and in the encouragement of the patient to reflect on present smoking behaviour in arguments against smoking and in informing the patient about benefits of smoking cessation. This included asking the patient to weigh the pros and cons of smoking cessation, offering assistance for smoking cessation, informing the patient about smoking cessation and the withdrawal process, providing information about Nicotine Replacement Therapy including instructions, and offering to discuss smoking again during the next visit (all *p* < 0.05).

For the most part, there was a notable increase in the approximate measure of the “sum of observed skills” for each student, as seen in the video-analysis pre- versus 4-weeks post-course as shown in Additional file 6: Figure S2 (from mean 9.1 (SD 2.0) to 11.8 (SD 2.2); *p* < 0.0005; Cohen’s d = 1.3).

#### Attitude

##### c1) Subjective (self-) assessment of “attitude”

General attitude towards smoking counselling changed dramatically in questions 1 and 4, as also evidenced by Cohen’s *d*. Questions 2 and 4 showed further changes between pre- and 4 weeks post-course, including a definite attitude to help smokers. Many students already expressed this attitude before the course, so there is probably some ceiling effect so that residual potential changes might only be small. Attitudes toward smokers as persons also became more tolerant, possibly reflecting more emotional understanding in their dependency; however, other very personal questions on the place of smokers in the students personal life did not change. Results are given in Additional file 7: Table S1.

##### c2) Externally assumed assessment of “attitude”

The objective assessment of students’ attitude showed a major positive change in virtually all assessed dimensions, as represented in Additional file 2: Table S2.

### Questions on students benefits on the visual analog scale (0 – 100)

In reply to the question whether they think that it would be possible to counsel smokers on the basis of the knowledge and skills learned in the course, most students answered that they agreed strongly with this statement (strongly agree = 0–19 pts; mean = 17.4; SD = 3.8).

All 87 students agreed that they would like to participate in further courses of the same style and with a similar goal setting (1 = agree; mean = 1.0; SD = 0.0). More items about the students’ evaluation after the course and their results are shown in Additional file 8.

## Discussion

The study confirms that a compact comprehensive 4-h interactive smoking cessation workshop for medical students is effective in terms of a profound short-term effect on the participants’ counselling abilities. Its design allows easy inclusion in the medical curriculum. As the course was designed, organized and conducted by medical students for their fellow medical students and supported by experts, its low costs makes it feasible for low- and middle income countries, where institutional receptivity for integration of tobacco control education in the medical curricula is frequently limited. The workshop was implemented “by students for students” thus promoting corporate culture and identity among medical students, which may have been part of the particularly high acceptance of the course. Furthermore, in their spontaneous feedback, many students considered this smoking cessation counselling workshop to be “in line with the trend.”

The quality of teaching during medical education is an important predictor of medical students’ learning outcomes [22]. Observations of community-based faculties indicated strong deficits in the provision for feedback [23]. In order to motivate students and to leave room for feedback, we offered the course to all medical students in the context of voluntary participation independent of their educational level. Their feedback confirmed that the course had a fundamental effect on “the enhancement of physician-patient communication” in general. Active educational methods, particularly standardized patient-physician role-playing were used in this study to foster “learning by doing” of counselling skills. Whereas the practical parts of the course emphasized brief intervention strategies [24, 25] using key components of Motivational Interviewing for the range of patients willing to quit - to patients unwilling to quit. The course was intended to form a focused, albeit expandable basis for clinical practice. Students were provided with the knowledge and skills needed to reasonably fill a minimal - but realistic - intervention time of approximately 5 min according to the patient’s individual stage of readiness to quit. The vast majority of students rated the exercises and intervention training educational content as highly important and appropriate for their level of training and for their future clinical work. This is also reflected by the fact that at the 4-week evaluation, 77% of the students had not consulted the hand-outs, underlining that the learning experience was, in effect, the course itself.

When and how to learn smoking cessation counselling?

In our study the “two-half day training program” suggested in Humair and colleagues “new curriculum to train residents in smoking cessation” [13] was reduced to a 4-h comprehensive course en bloc, and tailored to medical students’ educational needs independent of their academic year. Similar courses at 12 US medical schools [26] were quite heterogeneous, and lasted 4 to18 h.

By including education about tobacco in the medical curricula early on, students can be alerted to the health effects of tobacco use and learn to assist smokers to quit. This paper describes an efficient and economic setting for a smoking cessation course using the Stages-of-Change model by Prochaska, et al. [15] to teach medical students how to provide smoking cessation counselling tailored to the smoker’s individual readiness and motivation and to prescribe pharmacological therapy. The Transtheoretical Model is an evidence-based model of behavior change that has been developed and tested during the past decades in the context of smoking cessation [27]. Antypas and colleagues see the potential to combine the efficacy of health behavior change theory-based interventions with the higher perceived usefulness of interventions designed according to patient input [28].

In their anonymous feedback most students strongly agreed that the course provided not just the competence to approach and counsel smokers in a specifically scientific way, and overcome theoretical arbitrary dividing lines in order to assign patients to categories by means of a diagnosis and a consecutive treatment plan. They also learned about the physician-patient relationship in general, more or less detached from theoretical assessment tools, thus enabling them to expand and modify their counselling skills according to the individual needs of their future patients. The use of the Stages-of-Change model to behaviours other than tobacco dependence is an advantageous teaching method for promoting behavioural change in addiction in general, such as exercise, sleep hygiene, or nutrition, rather than specific techniques for specific behaviours [29].

For the purpose of an efficient training methodology and a proper evaluation of the issue of smoking cessation, competence was differentiated into the components “knowledge,” “skills” and “attitude,” which is well established in the literature [30]. Students demonstrated significant, and considering Cohen’s *d* effect size and student feedback, probably highly relevant improvements in key measurements of knowledge, skills and attitude regarding smoking cessation counselling and in the confidence and motivation to apply those competence elements in practice as future physicians. Medical students felt more knowledgeable and confident in following the recommended practices and offering cessation counselling to their future patients, as shown in Additional file 7: Table S1.

The most important outcome of knowledge, skill and attitude results may be the remarkable shift in attitude. Behaviour towards the patient is probably most influenced in the long-term by attitude.

### Study limitations

As there are no existing validating tools, therefore the questionnaires have not been validated. The current assessment for physicians and trainees formats reliably test core knowledge and basic skills. However, they may underemphasize some important domains of professional medical practice, including interpersonal skills, lifelong learning, professionalism, and the non-trivial step of integration of core knowledge and skills into clinical practice. In addition to assessments of basic skills, new formats that assess clinical reasoning, expert judgment, management of ambiguity, professionalism, time management, learning strategies, and teamwork would require optimal multidimensional assessments. Institutional support, reflection, and mentoring should accompany their development and change medical school programs and education.

Unfortunately, most program evaluations - ours included - lack long-term follow-up.

Variations in counselling competence between genders were not considered. Empirical findings on gender differences in smoking cessation with focus on 1) nicotine replacement therapy, 2) depression and anxiety factors, 3) post-cessation weight gain and body-shape concerns, 4) post-cessation withdrawal, and 5) the importance of social support during smoking cessation might play a role.

There may exist an important selection bias due to non-randomized selection of the participants. The course took place on a voluntary basis, and students who voluntarily enrolled for this course may be more motivated.

## Conclusions

We conclude that the study’s major objectives were achieved as significant and assumed relevant gains in medical students’ dimensions of competence (knowledge, skills, attitude) for successful patient-centred smoking cessation counselling could be measured. The students’ anonymous feedback was an indicator for the courses feasibility and for improved self-efficacy, suggesting that such a course was tailored to their competence needs, and could be implemented into medical school curricula. The course seems highly effective in promoting future physicians’ ability in smoking cessation counselling and thus in the long-term retention of medical students’ preventive medical competence. Although not shown, such trained competences may foster general counselling competences in further areas, such as addiction, nutrition, exercise and weight, stress management, or sleep issuescounselling could be measured.

## Additional files

Additional file 1: Definitions of Competence Dimensions. (DOCX 26 kb)
Additional file 2: Table S2.
*Externally Assumed Assessment of Attitude, Analyzed by the Two Standardized Patient Videos Pre- and 4 Weeks Post-Course (Visual Analog Scale [VAS] Results)*. (DOCX 18 kb)
Additional file 3: Evaluation Sheet for the filmed Role-Play. (DOCX 19 kb)
Additional file 4: Structure of the Evaluation Sheet. (DOCX 22 kb)
Additional file 5: Figure S1.
*Knowledge: Before and 4 Weeks After the Course*. (DOCX 16 kb)
Additional file 6: Figure S2.
*Skills of the Students: Two 5-min Standardized Videos Before and 4 Weeks After the Course*. (DOCX 19 kb)
Additional file 7: Table S1.
*Subjective, Questionnaire-Based (Self-) Assessment of Attitude Before and After the Course (Visual Analog Scale [VAS] Results). (DOCX 18 kb)*

Additional file 8: Anonymous Evaluation by the Participants 3 Days and 4 Weeks after the Course. (DOCX 20 kb)
