# Supplementary material for: Outcome of a four-hour smoking cessation counselling workshop for medical students
Source: Tob Induc Dis. 2016 Nov 25;14:37. doi: 10.1186/s12971-016-0103-x (PMC5123240; doi:10.1186/s12971-016-0103-x)
Supplement: Additional file 1: — Definitions of Competence Dimensions. (DOCX 26 kb) [file 12971_2016_103_MOESM1_ESM.docx]

**Additional file 1**

**Definitions of Competence Dimensions**

Knowledge

An evidence-based theoretical basis for models of practical strategies and algorithms of major steps for the successfully treatment of patients who smoke, according to national and international guidelines [1-4].

Skills

The physician’s medical and communicative competence and ability to balance between biomedical and psychosocial issues [5] in the context of this topic its relationship to patients.

Attitude

The sum of the clinicians’ affective, cognitive and behavioural intentions [6, 7] towards the smoking patient, tobacco dependence, and its treatment.

**References**

1. Torrijos RM, Glantz SA: The US Public Health Service "treating tobacco use and dependence clinical practice guidelines" as a legal standard of care. *Tob Control* 2006, 15:447-451.

2. Hays JT, Ebbert JO, Sood A: Treating tobacco dependence in light of the 2008 US Department of Health and Human Services clinical practice guideline. *Mayo Clin Proc* 2009, 84:730-735; quiz 735-736.

3. Lagrue G, Le Foll B, Melihan-Cheinin P, Rostoker G, Ades J, de Beaurepaire R, Berlin Y, Borgne A, Coninx P, Dautzenberg B, et al: [Clinical practice guideline: medical and nonmedical therapeutic strategies for smoking cessation. Bit of therapeutic practice: management and current practice in smoking cessation]. *Rev Mal Respir* 2003, 20:791-794.

4. Fiore M, Hatsukami D, Baker T: Effective tobacco dependence treatment. *JAMA* 2002, 288:1768-1771.

5. Roter DL, Stewart M, Putnam SM, Lipkin M, Jr., Stiles W, Inui TS: Communication patterns of primary care physicians. *JAMA* 1997, 277:350-356.

6. Kothandapani V: Validation of feeling, belief, and intention to act as three components of attitude and their contribution to prediction of contraceptive behavior. *J Pers Soc Psychol* 1971, 19:321-333.

7. Ostrom TM: The relationship between the affective, behavioral, and cognitive components of attitude. *Journal of Experimental Social Psychology* 1969, 5:12-30.
