# Supplementary material for: Outcome of a four-hour smoking cessation counselling workshop for medical students
Source: Tob Induc Dis. 2016 Nov 25;14:37. doi: 10.1186/s12971-016-0103-x (PMC5123240; doi:10.1186/s12971-016-0103-x)
Supplement: Additional file 2: Table S2. — Externally Assumed Assessment of Attitude, Analyzed by the Two Standardized Patient Videos Pre- and 4 Weeks Post-Course (Visual Analog Scale [VAS] Results). (DOCX 18 kb) [file 12971_2016_103_MOESM2_ESM.docx]

**Additional file 2**

**Table S2. *Externally Assumed Assessment of Attitude, Analyzed by the Two Standardized Patient Videos Pre- and 4 Weeks Post-Course (Visual Analog Scale [VAS] Results)***

| Item | *d* | % | *p* | Before | | After | |
| --- | --- | --- | --- | --- | --- | --- | --- |
|  |  |  |  | *M* | *SD* | *M* | *SD* |
| 1. The development of synchronicity in terms of head, body, and leg positioning | .99 | 13.0 | <.001** | 61.8 | 8.7 | 69.9 | 7.5 |
| 2. A creative and flexible style of speech | .60 | 7.6 | <.001** | 66.9 | 9.3 | 71.9 | 7.6 |
| 3. The counsellor’s ability to convey the meaning of personal responsibility for smoking | .63 | 7.9 | <.001** | 71.2 | 11.1 | 76.8 | 5.8 |
| 4. The ability of the counsellor to demonstrate an empathetic approach to the patients’ concerns and the smoking issue | .57 | 7.4 | <.001** | 68.9 | 10.5 | 74.0 | 7.3 |
| 5. The counsellor acts respectfully towards the patient | .67 | 7.0 | <.001** | 71.8 | 8.4 | 76.8 | 6.3 |
| 6. The counsellor’s sense of responsibility for the task of smoking cessation counselling | 1.0 | 14.3 | <.001** | 68.3 | 12.1 | 78.1 | 5.8 |
| 7. The atmosphere is relaxed | .51 | 7.1 | .001** | 68.7 | 11.7 | 73.6 | 6.8 |
| 8. The counsellor is able to involve the patient in the discussion | .54 | 8.8 | <.001** | 64.2 | 10.6 | 69.9 | 10.3 |
| 9. The patient can be proactive and take the initiative in the discussion | .17 | -1.9 | .264 | 73.4 | 7.1 | 72.0 | 9.1 |
| 10. The talk proportion of the patient (subjective perception) | .32 | -3.1 | .035* | 53.9 | 5.2 | 52.2 | 5.0 |
| 11. Extent to which the counsellor was able to implement relevant content in the interview | 1.3 | 20.4 | <.001** | 62.0 | 11.4 | 74.7 | 7.0 |
| 12. Assessment of the counsellor’s interpersonal relationship (compassion, patient centeredness, dignity) | .61 | 7.6 | <.001** | 69.9 | 9.3 | 75.2 | 8.0 |
| 13. This is an optimal interview | 1.2 | 19.0 | <.001** | 61.8 | 11.3 | 73.6 | 8.5 |

*Note*. % = Percent value of increase in VAS (VAS: 0 = strongly disagree, 100 = strongly agree [except item 10 where a symmetry in talking was 50% and total talking time by the patient was 100]); participants = 85.
