# Supplementary material for: Outcome of a four-hour smoking cessation counselling workshop for medical students
Source: Tob Induc Dis. 2016 Nov 25;14:37. doi: 10.1186/s12971-016-0103-x (PMC5123240; doi:10.1186/s12971-016-0103-x)
Supplement: Additional file 3: — Evaluation Sheet for the filmed Role-Play. (DOCX 19 kb) [file 12971_2016_103_MOESM3_ESM.docx]

**Additional file 3**

**Evaluation Sheet for the filmed Role-Play**

The counsellor has:

- recognized the patient’s nicotine dependence
- determined the daily amount of tobacco consumption
- determined the duration of tobacco consumption
- identified the motivation to quit smoking
- asked the patient about prior smoking cessation attempts
- identified the smokers’ degree of nicotine dependence: the period of time between awakening and the first cigarette
- addressed the advantages of smoking
- addressed the health risks of smoking
- encouraged the consideration of present smoking behaviour by underlining arguments against smoking
- encouraged the patient to weigh the pros and cons of smoking cessation
- discussed the obstacles to dishabituation: detoxification, stress, relapse
- noted personal information about smoking: health, family, finances
- recommended that the patient quit smoking
- provided assistance for dishabituation
- left the decision to quit smoking to the patient
- suggested practical strategies to facilitate smoking cessation
- discussed smoking cessation and the detoxification process
- talked about nicotine substitutes
- offered to discuss smoking again during the next visit
- arranged an appointment to speak about smoking cessation
