# Supplementary material for: Outcome of a four-hour smoking cessation counselling workshop for medical students
Source: Tob Induc Dis. 2016 Nov 25;14:37. doi: 10.1186/s12971-016-0103-x (PMC5123240; doi:10.1186/s12971-016-0103-x)
Supplement: Additional file 4: — Structure of the Evaluation Sheet. (DOCX 22 kb) [file 12971_2016_103_MOESM4_ESM.docx]

**Additional file 4**

**Structure of the Evaluation Sheet**

The rating schedule assessed students’ communicative skill performances and attitudinal changes on the videotaped interviews. The schedule covered important aspects of a smoking cessation intervention, including essential elements of brief motivational interviews [1-3] as indicated in the literature. The rating schedule has 2 sections with a total of 33 individual items.

Section 1 “Skills” (20 items) evaluated the extent that the students’ interviews covered a list of substantial contents for an “optimal” smoking cessation counselling technique, ranging from 1) identification of tobacco use; 2) encouragement to reflect on smoking behaviour; 3) offer of support smoking cessation (i.e., Ask, Advise and Assist). This section is shown in Appendix 2.

Finally the blinded independent evaluators had to ascertain whether the 5-minute video sequence of the recorded smoking cessation counselling was attended by the student prior to or after the course.

Section 2 (13 items) registered and measured the interviewer’s attitude in order to evaluate the course’s impact on attitudinal shifts, as well as e.g. harmonic interviewing style and body synchronicity: all of which are indispensable for an empathetic approach.

They were able to add written statements and observations concerning their decisions.

**References**

1. Foley K, George G, Crandall S, Walker K, Marion G, Spangler J: Training and evaluating tobacco-specific standardized patient instructors. *Fam Med* 2006, 38:28-37.

2. Rollnick S, Mason P, Butler C: *Health Behaviour Change: A Guide for Practitioners.* London: Churchill Livingstone; 1999.

3. Martino S, Haeseler F, Belitsky R, Pantalon M, Fortin At: Teaching brief motivational interviewing to Year three medical students. *Med Educ* 2007, 41:160-167.
