# Supplementary material for: Outcome of a four-hour smoking cessation counselling workshop for medical students
Source: Tob Induc Dis. 2016 Nov 25;14:37. doi: 10.1186/s12971-016-0103-x (PMC5123240; doi:10.1186/s12971-016-0103-x)
Supplement: Additional file 5: Figure S1. — Knowledge: Before and 4 Weeks After the Course. (DOCX 16 kb) [file 12971_2016_103_MOESM5_ESM.docx]

**Additional file 5**

**Figure S1: *Knowledge: Before and 4 Weeks After the Course***

**P**aired samples t-test with 85 cases: “Knowledge” before and four weeks after the course; mean difference 8.7 (95%CI = 7.8 to 9.6; p < 0.0005); Cohen’s d = 2.7.

Accordingly to Cohen’s d of 2.7 the magnitude shows a significant effect.
