# Supplementary material for: Outcome of a four-hour smoking cessation counselling workshop for medical students
Source: Tob Induc Dis. 2016 Nov 25;14:37. doi: 10.1186/s12971-016-0103-x (PMC5123240; doi:10.1186/s12971-016-0103-x)
Supplement: Additional file 6: Figure S2. — Skills of the Students: Two 5-min Standardized Videos Before and 4 Weeks After the Course. (DOCX 19 kb) [file 12971_2016_103_MOESM6_ESM.docx]

**Additional file 6**

**Figure S2: *Skills of the Students: Two 5-Minute Standardized Videos Before and 4 Weeks After the Course***

__

Paired samples t-test with 85 cases: “Sum of skills” before and four weeks after the course; mean difference -2.7 (95%CI = -3.4 to -2.1; p < 0.0005); Cohen’s d = 1.3. This Cohen’s d of 1.3 shows a significant effect.
