# Supplementary material for: Outcome of a four-hour smoking cessation counselling workshop for medical students
Source: Tob Induc Dis. 2016 Nov 25;14:37. doi: 10.1186/s12971-016-0103-x (PMC5123240; doi:10.1186/s12971-016-0103-x)
Supplement: Additional file 7: Table S1. — Subjective, Questionnaire-Based (Self-) Assessment of Attitude Before and After the Course (Visual Analog Scale [VAS] Results). (DOCX 18 kb) [file 12971_2016_103_MOESM7_ESM.docx]

**Additional file 7**

**Table S1. *Subjective, Questionnaire-Based (Self-) Assessment of Attitude Before and After the Course (Visual Analog Scale [VAS] Results)***

| Item | *d* | % | *p* | *N* | Before | | After | |
| --- | --- | --- | --- | --- | --- | --- | --- | --- |
|  |  |  |  |  | *M* | *SD* | *M* | *SD* |
| **a) General Attitude Towards Smoking Counselling** |  |  |  |  |  |  |  |  |
| 1. Counselling smokers is highly significant. | .56 | 11.9 | <.0001** | 87 | 81.5 | 18.2 | 91.1 | 16.4 |
| 2. Nicotine dependency is an addiction. I am not a psychiatrist. I am not responsible for assessing patients’ smoking status if they do not specifically ask for a smoking cessation interview. | .39 | -41.8 | .004* | 88 | 16.2 | 18.3 | 9.4 | 16.3 |
| 3. Nicotine dependency is an addiction. If a patient has a heart attack, I have to be engaged in the smoking issue. | .23 | 3.5 | .111 | 87 | 89.4 | 14.8 | 92.6 | 12.6 |
| 4. As a (future) physician I believe it is necessary to assess all patients concerning their smoking status and to mention potential problems and possibilities for intervention. | .89 | 35.6 | <.0001** | 88 | 60.9 | 29.3 | 82.6 | 17.9 |
|  |  |  |  |  |  |  |  |  |
| **b) Attitude Towards Smokers** |  |  |  |  |  |  |  |  |
| 1. I am tolerant towards smokers. | .39 | 23.8 | <.0001** | 88 | 49.6 | 31.1 | 61.4 | 30.1 |
| 2. Among my friends I like smokers the same as non-smokers. | .08 | -2.3 | .377 | 88 | 83.3 | 22.4 | 81.4 | 25.0 |
| 3. I have no preference between dating a smoker or a non-smoker. | .01 | 0.6 | .917 | 88 | 47.3 | 38.3 | 47.6 | 34.6 |
| 4. Smokers are rather weak and have less volition. | .06 | 4.9 | .521 | 88 | 30.9 | 24.9 | 32.4 | 28.3 |
| 5. Smokers are more ruthless. | .13 | -6.8 | .210 | 88 | 53.2 | 28.0 | 49.6 | 27.1 |
| 6. Smokers are self-inflicting, so I will not advise them. | .14 | -18.3 | .297 | 88 | 8.5 | 12.2 | 6.9 | 10.8 |
| 7. Smokers are self-inflicting, but I will help them anyway. | .21 | -7.0 | .081 | 88 | 77.5 | 23.0 | 72.1 | 29.5 |
|  |  |  |  |  |  |  |  |  |
| **c) Own Attitude to Smoking Behaviour – (If a Smoker)** |  |  |  |  |  |  |  |  |
| 1. This course has a bearing on my own smoking behaviour. | - | 28.5 | .097 | 40 | 37.8 | - | 48.6 | - |

*Note.* % = Percent change of the mean between pre/post; VAS: 0 = strongly disagree, 100 = strongly agree.

The most important outcome of knowledge, skill and attitude results may be this significant shift in general attitude towards smoking counselling with a median Cohen’s d of .52. This probably influences the long-term effect, as e. g. on behaviour towards the patient.
