# Supplementary material for: Outcome of a four-hour smoking cessation counselling workshop for medical students
Source: Tob Induc Dis. 2016 Nov 25;14:37. doi: 10.1186/s12971-016-0103-x (PMC5123240; doi:10.1186/s12971-016-0103-x)
Supplement: Additional file 8: — Anonymous Evaluation by the Participants 3 Days and 4 Weeks after the Course. (DOCX 20 kb) [file 12971_2016_103_MOESM8_ESM.docx]

**Additional file 8**

**Anonymous Evaluation by the Participants 3 Days and 4 Weeks after the Course**

87 of 88 participants completed the evaluation form.

**Course Content and Participant Benefits**

| Item | *M* | *SD* |
| --- | --- | --- |
| The participants felt that they had increased their on-topic knowledge after the course. | 8.8 | 6.2 |
| The participants found that the topic of “smoking” was treated fairly and reasonably in the course. | 8.8 | 6.2 |
| The participants thought that they increased their knowledge about the physician-patient relationship. | 19.3 | 13.7 |
| The participants found it would be possible to counsel smokers on the basis of the knowledge and skills they learned in the course. | 17.4 | 3.8 |

*Note.* Scale 0-19 pts = strongly agree; *M* = Mean; *SD* = Standard Deviation.

| Item | *M* | *SD* |
| --- | --- | --- |
| The participants benefited from the course concerning their skills in approaching patients and counselling smokers. | 83.7 | 11.5 |
| The participants benefited from the course in their attitude and motivation to integrate smoking cessation counselling in their future clinical work. | 76.9 | 16.3 |
| The participants thought that they were more likely to apply smoking cessation counselling after the course. | 51.8 | 34.1 |

*Note.* Scale: 40-59 pts = rather yes, 60-79 pts = agree, 80-100 pts = strongly agree; *M* = Mean; *SD* = Standard Deviation.

**Teaching Methods**

Most students agreed that the power point presentation was clear and well-structured (Mean= 3.3; SD= 0.5), and contained very valuable information (Mean= 3.6; SD= 0.3). As an aid to facilitate practical training in role-playing, some of the content of the flip chart was projected onto a screen. The majority of the participants rated the projected slides to be “appropriate and helpful” (Mean= 3.2; SD= 0.6). More than 70% of the students “strongly agreed” that the flip chart was “appropriate and helpful” for the course (Mean= 3.4; SD= 0.4). Almost 60% of the students “strongly agreed” that the reference manual was “appropriate and helpful” (Mean= 3.1; SD= 0.7). Most participants felt that adequate time was allowed for role playing (Mean= 3.4; SD= 0.3), and that its the duration was adequate (0 = too short, 100 = too long, mean= 44.6; SD= 3.8). The evaluation also assessed how much (0-100%) of the reference manual was “paged through” and “effectively read” by the students in the time between pre- and post-course assessment, as the course was scheduled for 3-4 week intervals to allow acquisition of on-topic knowledge between sessions. 77% of the participants did not page through the reference manual at all (0%= 0-19 pts; Mean= 11.6; SD= 5.4), and about 21% paged through less than 50% of the script. 82 out of 87 participants “effectively read” 0% of the reference manual (0%=0-19 pts; Mean= 4.4; SD= 3.1). The majority of the students found the script to be rather a valuable tool as a “work of reference” for the present and future clinical practice (strongly agree= 0-19 pts; Mean= 16.6; SD=2.4).

**Support and Feedback by the Supervising Expert Team**

A majority of the students agreed that any questions were clearly answered by the supervisors (Mean= 3.6; SD= 0.3), and found that “support and feedback” provided by the supervisors was “very good” (0-19 pts) (Mean= 18.9; SD= 1.5).

**Course Structure**

The majority of participants found the structure of the course to be “very good” (0-19 pts) (Mean=13.0; SD= 9.2). To the question “What is your opinion of the course?” all students agreed that the course was “good” or “very good” (Very good= 0-19 pts; Mean= 9.2; SD= 6.5).

**General Framework and Conditions of the Course**

When given the occasion to evaluate the timeframe (day of week, time of day) all students preferred a Friday course in the afternoon to a weekend course on a Saturday morning (Saturday=0; Friday=1; Mean= 1; SD= 0.0). Students were highly satisfied with the courses’ provisions and beverages (very good = 0-19 pts; Mean= 5.6; SD= 4.0). Technical equipment was rated to be adequate (Mean= 3.5; SD= 1.0).

The need for such a course was also underlined by the spontaneous feedback of the students before, during and after the courses. More than half of the participants (57%) felt motivated and confident enough to conduct such a course by themselves (rather yes= 40-59 pts; Mean= 53.4; SD= 7.4).

**References**

1. Torrijos RM, Glantz SA: The US Public Health Service "treating tobacco use and dependence clinical practice guidelines" as a legal standard of care. *Tob Control* 2006, 15:447-451.

2. Hays JT, Ebbert JO, Sood A: Treating tobacco dependence in light of the 2008 US Department of Health and Human Services clinical practice guideline. *Mayo Clin Proc* 2009, 84:730-735; quiz 735-736.

3. Lagrue G, Le Foll B, Melihan-Cheinin P, Rostoker G, Ades J, de Beaurepaire R, Berlin Y, Borgne A, Coninx P, Dautzenberg B, et al: [Clinical practice guideline: medical and nonmedical therapeutic strategies for smoking cessation. Bit of therapeutic practice: management and current practice in smoking cessation]. *Rev Mal Respir* 2003, 20:791-794.

4. Fiore M, Hatsukami D, Baker T: Effective tobacco dependence treatment. *JAMA* 2002, 288:1768-1771.

5. Roter DL, Stewart M, Putnam SM, Lipkin M, Jr., Stiles W, Inui TS: Communication patterns of primary care physicians. *JAMA* 1997, 277:350-356.

6. Kothandapani V: Validation of feeling, belief, and intention to act as three components of attitude and their contribution to prediction of contraceptive behavior. *J Pers Soc Psychol* 1971, 19:321-333.

7. Ostrom TM: The relationship between the affective, behavioral, and cognitive components of attitude. *Journal of Experimental Social Psychology* 1969, 5:12-30.

8. Foley K, George G, Crandall S, Walker K, Marion G, Spangler J: Training and evaluating tobacco-specific standardized patient instructors. *Fam Med* 2006, 38:28-37.

9. Rollnick S, Mason P, Butler C: *Health Behaviour Change: A Guide for Practitioners.* London: Churchill Livingstone; 1999.

10. Martino S, Haeseler F, Belitsky R, Pantalon M, Fortin At: Teaching brief motivational interviewing to Year three medical students. *Med Educ* 2007, 41:160-167.
